# Supplementary material for: Human Colonoid–Myofibroblast Coculture for Study of Apical Na+/H+ Exchangers of the Lower Cryptal Neck Region
Source: Int J Mol Sci. 2023 Feb 21;24(5):4266. doi: 10.3390/ijms24054266 (PMC10001859; doi:10.3390/ijms24054266)
Supplement: Supplementary file 1 [file ijms-24-04266-s001.zip › ijms-2124932-supplementary.pdf]

International Journal of
Molecular Sciences

MDPI

Article

Human Colonoid–Myofibroblast Coculture for Study of Apical Na^+/H^+ Exchangers of the Lower Cryptal Neck Region

Azam Salari [1], Kunyan Zhou [1,2], Katerina Nikolovska [1], Ursula Seidler [1,*] and Mahdi Amiri [1,*]

[1] Department of Gastroenterology, Hepatology and Endocrinology, Hannover Medical School, 30625 Hannover, Germany
[2] Department of Thyroid Surgery, The First Affiliated Hospital, School of Medicine, Zhejiang University, Hangzhou 310027, China
* Correspondence: seidler.ursula@mh-hannover.de (U.S.); amiri.mahdi@mh-hannover.de (M.A.); Tel.: +49-511-532-9427; Fax: +49-511-532-8428 (U.S.)

Supplementary Materials

Table S1. *Sequence of primers used for RT-qPCR experiments.*

Gene	Forward Primer	Reverse Primer
ACTB	CGAGGACTTTGATTGCACATTGTT	TGGGGTGGCTTTTAGGATGG
AE2	TGCCAAAGGTTCCACACA	CAACTCATTCAGCTCCACAAAC
ALPI	CGCTTTAACCAGTGCAACAC	GTCACCACTCCTACTGACTTTC
CFTR	CTACCACTGGTGCATACTCTAATC	ACGTGTTGAGGGTTGACATAG
CLDN1	CTGGGAGGTGCCCTACTTTG	ACACGTAGTCTTTCCCGCTG
CLDN2	GATCCTACGGGACTTCTACTCA	CAGGGAGAACAGGGAAGAAATAA
CLDN3	ACGCGAGAAGAAGTACACGG	TAGACGTAGTCCTTGCGGTC
CLDN4	TTCTACAATCCGCTGGTGGC	GCGGAGTAAGGCTTGTCTGT
CLDN7	GGGGGAGACGACAAAGTGAA	CATACCAGGAGCAAGCTACCA
CLDN8	TGCCCAAAAACGTGAGCTTG	TGTGCGATGGGAAGGTATCG
GAPDH	TGCACCACCAACTGCTTAGC	GGCATGGACTGTGGTCATGAG
GREM1	GCAAGCCCAAGAAATTCACTAC	TGCAACGACACTGCTTCA
KI67	GACCTCAAACTGGCTCCTAATC	GCTGCCAGATAGAGTCAGAAAG
LGR5	CCTGCTTGACTTTGAGGAAGACC	CCAGCCATCAAGCAGGTGTTCA
LYZ	GGCTTGTCCTCCTTTCTGTTA	GTAGCCATCCATTCCCAATCT
MUC2	AGTTTGGGGAGCACTTCGAG	TCTTCCACGCAGTGGGTAAC
NBCe1	CCGGCTTTGTTGGTCACTAT	CAAGTGATACCCTGCTCCTTTC
NBCn1	CTGCTATTCCTGCTTTGCTTTG	GTGATAGCCAGCTCCTTTCTT
NHE1	GCTGGTGGCAGACCCCTACGA	ATAGGCCAGTGGGTCTGAGCCGA
NHE2	TGTCTACCGTGGGCAAGAAC	AACGCAAAACAGATGGCACC
NHE3	ACCGTGCGCTACACCATGAAGATG	ATGCGGTAGCGGTTCAGAAGCC
NHE8	CATGTGTGTTTGCATTTCTTGGC	AGCACTATGCACCAGATGACA
NKCC1	AAAGGAACATTCAAGCACAGC	CTAGACACAGCACCTTTTCGTG
OCLN	TCGACCAATGCTCTCTCAGC	CTCCTGGAGGAGAGGTCCAT
PDGFRA	CTTTGTCAGTGTTGCCTCTTG	GTTCACCTTCTGTGGCCTATTA
RSPO3	GAAAGAGGAGAAAGGAAGGGAAG	GGCTGCCGATGTATTCCATAA
SI	CGCTACACCTTATTACCCTTCC	CCAGCTGTTCGTATCCTCATAAA
SLC26A3	CCAGCGTCTATTCCCTCAAAT	TCCCAGCAAATCCTCTGAATAC
SLC26A6	AGAAACTGCTCAAGAAGCAGGA	CCATCTTATCTCCTGAGCTCACC
THY1 (CD90)	TGACCCGTGAGACAAAGAAG	GCTAGTGAAGGCGGATAAGTAG
WNT2B	CCTTGGAGTGGTAGCCATAAG	AACGCTGACTGTGTAGGTATG
WNT5a	GTGATGCAGATAGGCAGCCG	GCCATAGTCGATGTTGTCGC
ZO-1	CCTGAGTTTGACAGTGGAGTT	GCTGAAGGACTCACAGGAATAG

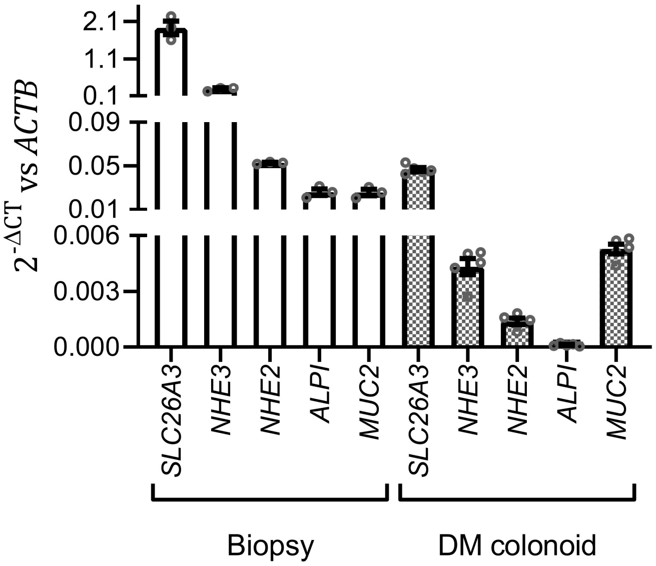

Figure S1. *SLC26A3* mRNA is highly expressed in human colon. RT-qPCR analysis of human transverse colon biopsy material from which the colonoids were derived shows that among the selected panel of genes, *SLC26A3* has substantially higher expression levels. Similarly in differentiated colonoid monolayers, *SLC26A3* expression is drastically higher than other differentiation marker genes including *NHE3*, *MUC2* or *ALPI*.

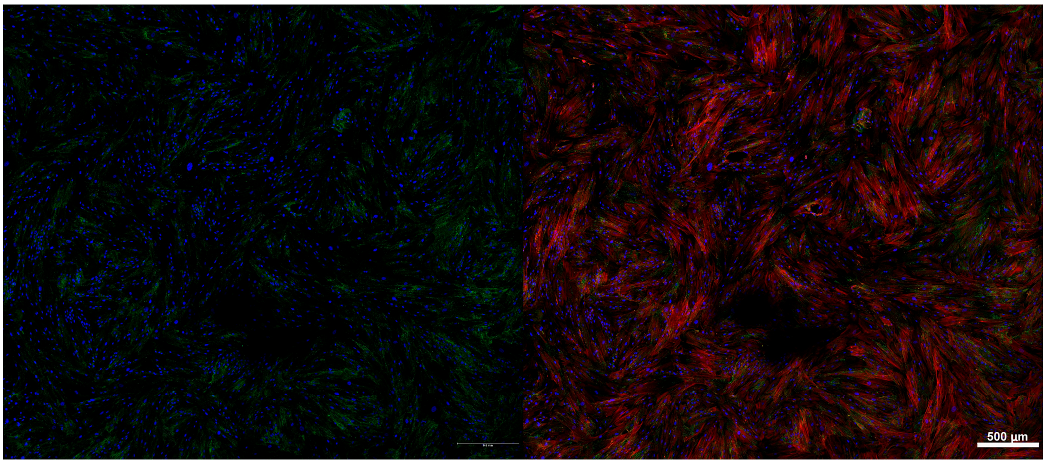

Figure S2. Majority of the cells in the myofibroblast culture used in CM-CE cocultures are CD90-positive. This immunofluorescent image shows intestinal myofibroblasts from the lamina propria of human transverse colon stained for nuclei (blue), CD90 (green) and F-actin (red). The image is generated by stitching multiple tile scan acquisitions to cover a broad area of the culture. The left and right panels are identical, expect that in the left panel the F-actin signal is excluded for a better visualization of the CD90 signal. The majority of the myofibroblasts are positively stained for CD90.

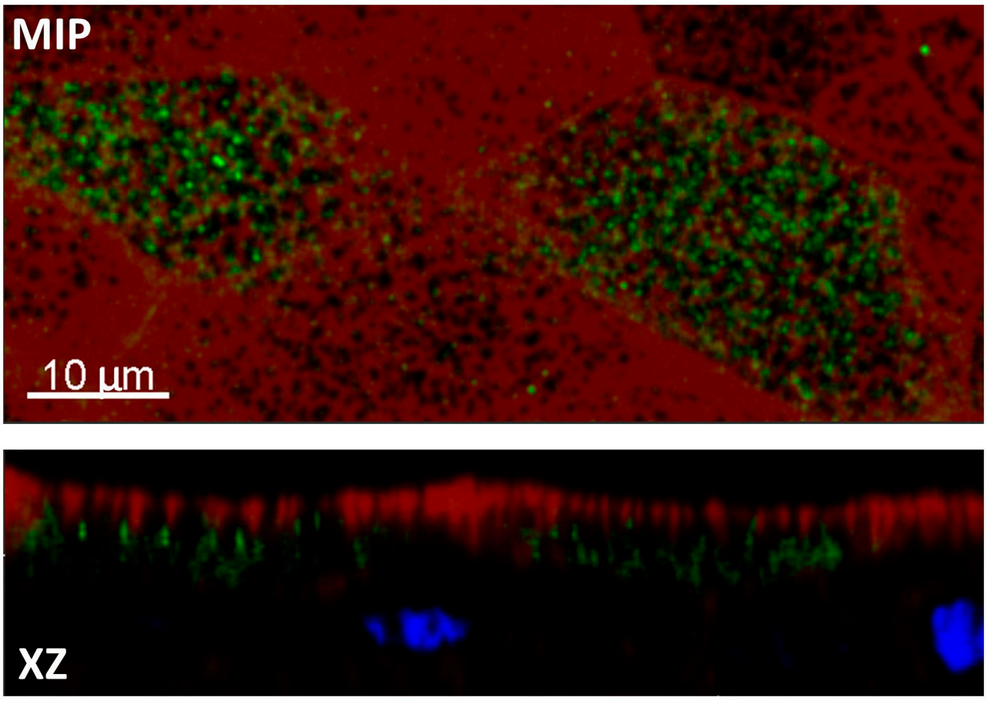

Figure S3. immunohistochemical staining of NHE8 in human CM-CE monolayers. Maximum intensity projection (MIP) and XZ cross section of CM-CE monolayer. NHE8 is detected as organellar structure mainly distributed between nucleus and subapical region. Green: NHE8, Red: F-actin, Blue: nuclei (excluded in MIP).
